# The fate of sulfonamide resistance genes and anthropogenic pollution marker *intI1* after discharge of wastewater into a pristine river stream

**DOI:** 10.3389/fmicb.2023.1058350

**Published:** 2023-01-25

**Authors:** Sarah Haenelt, Gangan Wang, Jonas Coelho Kasmanas, Florin Musat, Hans Hermann Richnow, Ulisses Nunes da Rocha, Jochen A. Müller, Niculina Musat

**Affiliations:** ^1^Department of Isotope Biogeochemistry, Helmholtz Centre for Environmental Research, Leipzig, Germany; ^2^Department of Environmental Microbiology, Helmholtz Centre for Environmental Research, Leipzig, Germany; ^3^Department of Molecular Biology and Biotechnology, Faculty of Biology and Geology, Babeş-Bolyai University, Cluj-Napoca, Romania; ^4^Isodetect Umweltmonitoring GmbH, Leipzig, Germany; ^5^Institute for Biological Interfaces (IBG 5), Karlsruhe Institute of Technology, Eggenstein-Leopoldshafen, Germany

**Keywords:** class 1 integron, sulfamethoxazole, sulfonamide resistance, *sul1*, *sul2*, *intI1*, river ecosystem, one health

## Abstract

**Introduction:**

Currently there are sparse regulations regarding the discharge of antibiotics from wastewater treatment plants (WWTP) into river systems, making surface waters a latent reservoir for antibiotics and antibiotic resistance genes (ARGs). To better understand factors that influence the fate of ARGs in the environment and to foster surveillance of antibiotic resistance spreading in such habitats, several indicator genes have been proposed, including the integrase gene *intI1* and the sulfonamide resistance genes *sul1* and *sul2*.

**Methods:**

Here we used quantitative PCR and long-read nanopore sequencing to monitor the abundance of these indicator genes and ARGs present as class 1 integron gene cassettes in a river system from pristine source to WWTP-impacted water. ARG abundance was compared with the dynamics of the microbial communities determined *via* 16S rRNA gene amplicon sequencing, conventional water parameters and the concentration of sulfamethoxazole (SMX), sulfamethazine (SMZ) and sulfadiazine (SDZ).

**Results:**

Our results show that WWTP effluent was the principal source of all three sulfonamides with highest concentrations for SMX (median 8.6 ng/l), and of the indicator genes *sul1*, *sul2* and *intI1* with median relative abundance to 16S rRNA gene of 0.55, 0.77 and 0.65%, respectively. Downstream from the WWTP, water quality improved constantly, including lower sulfonamide concentrations, decreasing abundances of *sul1* and *sul2* and lower numbers and diversity of ARGs in the class 1 integron. The riverine microbial community partially recovered after receiving WWTP effluent, which was consolidated by a microbiome recovery model. Surprisingly, the relative abundance of *intI1* increased 3-fold over 13 km of the river stretch, suggesting an internal gene multiplication.

**Discussion:**

We found no evidence that low amounts of sulfonamides in the aquatic environment stimulate the maintenance or even spread of corresponding ARGs. Nevertheless, class 1 integrons carrying various ARGs were still present 13 km downstream from the WWTP. Therefore, limiting the release of ARG-harboring microorganisms may be more crucial for restricting the environmental spread of antimicrobial resistance than attenuating ng/L concentrations of antibiotics.

## Introduction

The environmental fate of antimicrobial resistance is a crucial component of the One Health concept (McEwen and Collignon, 2018; The World Health Organization, The Food and Agriculture Organization of the United Nations, and The World Organisation for Animal Health, 2019). Surface waters are among the environmental reservoirs and potential dissemination avenues for antibiotic resistant bacteria and antimicrobial resistance genes (ARGs; Larsson and Flach, 2022). The close connection between such water bodies, animals, and humans entails the risk of cross-transmission, which ultimately jeopardizes the successful treatment of infectious diseases with antibiotics (Collignon et al., 2018). Therefore, monitoring the fate of ARGs in surface waters is an important component in assessing the risk of antimicrobial resistance spread in the environment to human and animal health (Berendonk et al., 2015; Wang et al., 2021).

The fate of ARGs in surface waters and other environmental compartments is likely influenced by their frequent association with mobile genetic elements such as transposons and plasmids (Yao et al., 2022). Such elements may promote ARG transfer from antibiotic resistant bacteria stemming from humans and warm-blooded animals to riverine microorganisms, eventually leading to the spread of resistance among aquatic bacterial communities (Koczura et al., 2016; Chaturvedi et al., 2021a). The mobility of ARGs can be further enhanced when they are associated with integrons, which are powerful tools for microorganisms to adapt quickly to changes in their environment (Mazel, 2006). Integrons can be located on mobile genetic elements or on the chromosome. They can acquire ARGs, heavy metal and disinfectant resistance genes (Gillings, 2018). The class 1 integron, the best-studied integron variant, consists of two conserved segments flanking a variable region (Supplementary Figure S1). The 5′ conserved segment (5′CS) contains the *intI1* gene, encoding for an integrase enzyme, an *attI* recombination site, and a promoter governing the expression of genes cassettes in the variable region. Gene cassettes or any DNA fragment carrying an *attC* recombination site can be inserted into or excised from the variable region by the integrase. The insertion mechanism can lead to gene cassette arrays, making the class 1 integron a carrier of multi-resistance (Cambray et al., 2010). The 3′ conserved segment (3′CS) harbors the *qacEΔ1* gene, encoding for resistance against quaternary ammonium compounds, and the *sul1* gene, conferring sulfonamide resistance. The relative abundance of the so-called clinical *intI1* variant of class 1 integron has increased in the natural environment worldwide due to human activities, including wastewater discharge and selection pressure from antibiotic use (Gillings et al., 2008). The *in situ* copy number of this gene is used as a proxy for anthropogenic pollution in the environment (Gillings et al., 2015). Given the co-occurrence of *intI1* and *sul1* in the class 1 integron, the abundance of these genes shows frequently a strong positive correlation in impacted environments (Gillings et al., 2015). Like *intI1*, *sul1* and its close relative *sul2* are used as indicators for the level of pollution with antimicrobial agents in the environment (Pruden et al., 2006; Adelowo et al., 2018; Lee et al., 2021; Chaturvedi et al., 2021b). Both *sul* genes are often located on mobile genetic elements such as plasmids, which may promote their dissemination in the environment (Chaturvedi et al., 2021b).

Prominent interfaces between human activities and surface waters are wastewater treatment plants (WWTPs), whose effluents increase the amounts of ARGs and antibiotic resistant bacteria in receiving waterbodies (Hernández et al., 2019; Wang et al., 2021). WWTPs also discharge a measurable amount of antibiotics into the aquatic environment. Sulfonamides like sulfamethoxazole (SMX) are commonly detected in WWTP effluents with average concentrations ranging from tens to hundreds of ng/L, while peak concentrations can even be in the low μg/L range (Carvalho and Santos, 2016). In the absence of extreme environmental conditions, they are chemically rather stable in surface waters (Straub, 2016). Together with their continuous presence in WWTP discharge, this leads to typical mean concentrations in receiving water bodies in the low ng/L range, with occasionally low μg/L concentrations (Martin-Laurent et al., 2019). It is an important question whether continuous sulfonamide contamination affects the spread of the corresponding ARGs and microbial community structure in riverine ecosystems and at what concentrations such effects are detectable. According to the European Committee on Antimicrobial Susceptibility Testing (EUCAST), the lowest minimal inhibitory concentration MIC for SMX in any microbial species is around 1 mg/l, thus 10^4^–10^6^ times higher than typical mean concentrations in surface waters (Bengtsson-Palme and Larsson, 2016). However, recent studies have also evaluated the predicted no-effect concentration (PNEC) for selection of sulfonamide resistance. The PNEC varies between 0.6 and 16 μg/l, with the first one being at the high end of SMX concentrations found in WTTP effluents (Bengtsson-Palme and Larsson, 2016; Le Page et al., 2017). In a recent study conducted by Borsetto et al. (2021), the effect of SMX on the microbiome of a WWTP-impacted river was tested over 24 days using an artificial river water flume system. The addition of 4 μg/l SMX did not lead to the spread of sulfonamide resistance genes in this particular experimental set-up. However, a potential draw-back of such experiments with a microbial community already influenced by WWTP effluent containing sulfonamides and other antimicrobials is, that short-term concentration increases may not generate detectable effects. Therefore, it remains uncertain whether SMX has an impact on riverine microbial communities at typical surface water concentrations, including whether it promotes the spread of ARGs in the environment (Cairns et al., 2018).

Here, we investigated how the microbial community of a pristine river is affected by WWTP effluent containing sulfonamides and the corresponding ARGs. As a model site, we selected the Holtemme river, which flows from the Harz mountains to the central German plain and has been well characterized within the Terrestrial Environmental Observatories (TERENO) (Zacharias et al., 2011; Wollschläger et al., 2017). We profiled the microbial community, monitored the abundance of *sul1*, *sul2* and *intI1*, and determined sulfonamide concentrations along the gradient of anthropogenic impact on the river. Furthermore, we carried out long-read nanopore sequencing of gene cassettes of the clinical class 1 integron in river samples to better understand how the integron contributes to the local prevalence of ARGs.

## Materials and methods

### Study region

This study was conducted on the Holtemme river in Saxony-Anhalt (Germany), which is serving as a landscape model for studies on the effects of anthropogenic pollution on riverine ecosystem functioning (Inostroza et al., 2016; Beckers et al., 2018; Brase et al., 2018). The third order river is 47 km long, originating from the low mountain range of Harz (862 m a.s.l), and drains a catchment area of 278 km^2^. After leaving the Harz national park, the river unites with a small stream called Zillierbach and passes through the city of Wernigerode (32,000 inhabitants), farmed plains, some smaller villages and the city of Halberstadt (39,000 inhabitants). There are two activated sludge-based, tertiary WWTPs, treating urban wastewater and discharging into the Holtemme, one just downstream Wernigerode (WWTP Silstedt) and one downstream Halberstadt. Two small brooks enter the Holtemme upstream and three more downstream from the WWTP Silstedt, all of which contribute very little to the water volume of Holtemme river. Downstream the mountainous region, the Holtemme is often rectified and canalized (Karthe et al., 2017). The land use in the river catchment is dominated by agriculture (60% of the catchment area), followed by forests (30%), and urban areas (10%). The above mentioned features render the Holtemme an excellent model system for studying the effect of anthropogenic pollutions on a pristine river ecosystem (Wollschläger et al., 2017).

### Water sampling and sample preparation

**Figure 1:**
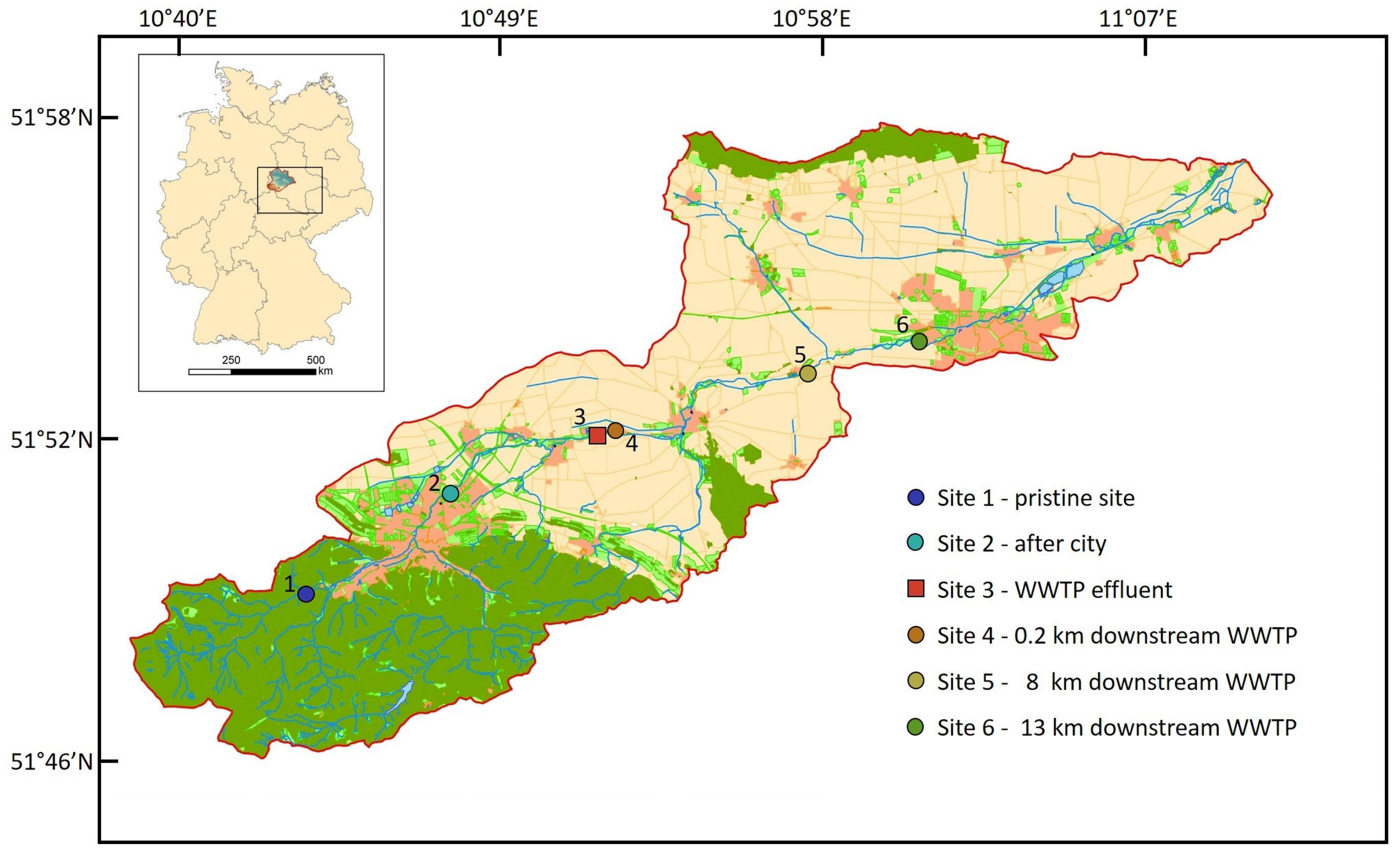
Location of all six sites in the catchment of the Holtemme river in the Harz mountain region, Germany. Map created from Authorative Topographic–Cartographic Information System (ATKIS).

Surface water samples were collected at six different Sites along the river stream (Figure 1). Site 1 (51°49′00.9″N 10°43′28.6″E) in the mountain region is a pristine Site with minor human impact. Site 2 (51°50′49.6”N 10°47′29.3″E) is located in the city of Wernigerode, Site 3 (51°51′59.4″N 10°51′43.3″E) is the discharge water of WWTP Silstedt, whereas Site 4 (51°51′59.0″N 10°51′55.5″E) is located 250 m downstream the WWTP. Site 5 (51°53′05.8″N 10°57′47.5″E) and Site 6 (51°53′46.5″N 11°01′03.5″E) are 8 and 13 km downstream Site 3 before the Holtemme enters the city of Halberstadt. Site 1–5 were sampled on December 7, 2020, and weekly from January 4 through February 1, 2021. Additional daily samples were collected from Site 1 to 6 from February 24 through March 4, 2021. Apart from eight unusually cold days in February 2021, precipitation and temperature patterns were representative for a winter season in this region (Supplementary Figure S2). No sampling was conducted during the unusually cold period in February 2021. For Sites 1 and 5, water flow rates are available from official measurement points of the state Saxony-Anhalt. At Site 1, rates varied between 0.063 and 0.119 m^3^/s with an average of 0.1 m^3^/s, and at Site 5 the average flow rate was 1 m^3^/s. At Site 3 (WWTP effluent) it ranged between 0.104 and 0.19 m^3^/s during the sampling period and values were provided by the WWTP Silstedt.

Water samples were collected in sterile 1 l glass flasks after pre-rinsing with river water by immersing the bottles into the river at a water depth of approximately 20 cm. Water samples were collected in triplicates at each Site. Afterwards, the samples were transported to the laboratory within 3 h in a thermostable box. At Sites 1 and 5, physicochemical parameters pH, water temperature [°C], conductivity [mS/cm] and ${\mathrm{NO}}_{3}^{-}$ -N [mg/L] were obtained in the MOBICOS monitoring stations located at the river (Fink et al., 2020). Physicochemical parameters from Site 3 (WWTP effluent) were kindly provided by the WWTP Silstedt.^1^ At Sites 2, 4, and 6, pH and temperature were measured using a SenTix41 probe (Xylem Analytics, Germany). Immediately after arriving in the laboratory, samples were prepared for further analyses. A volume of 500 ml of water was filtered (PSE PALL filters, diameter 47 mm, pore size 0.22 μm, Pall Corporation, NY United States) using a vacuum pump. Three filters per sample were prepared. One filter was used for DNA extraction and two were frozen at −20°C as back-up. For each sample, 100 ml of flow-through were collected and stored at 4°C overnight for the measurement of sulfonamide concentration.

### Solid phase extraction (SPE) and HPLC-MS analysis

Sulfamethoxazole (SMX), sulfadiazine (SDZ) and sulfamethazine (SMZ) concentrations were measured in seven replicates per Site (Supplementary Table S1). To concentrate the water samples, solid phase extraction was carried out using Oasis HLB 6 cc-500 mg columns (Waters, CT United States). Cartridges were conditioned with 4 ml of methanol (Chemsolute, Germany) and equilibrated with 4 ml of H_2_O (Chemsolute, Germany). Filtered river samples (100 ml) were loaded onto the column at a flow rate of 1 ml/min using a vacuum pump. After sample loading was completed, cartridges were dried for 5 min under vacuum. Analytes were eluted with 4 ml of methanol. Eluents were evaporated under a gentle stream of pure nitrogen until complete dryness. The analytes were then re-dissolved in 900 μl of H_2_O with 0.1% (*v*/*v*) formic acid (Serva, Germany) and 100 μl of methanol and transferred to HPLC vials. Vials were kept at −20°C until measurement at HPLC–MS.

For HPLC–MS analysis a stock solution of 10 mg/l mixed standard with SMX (Sigma-Aldrich, MO United states), SDZ (Alfa Aesar, MA USA) and SMZ (Sigma-Aldrich, MO, United states) was prepared in pure methanol and stored at −20°C. For the calibration curve, working concentrations of 0.01, 0.025, 0.05, 0.075, 0.1, 0.25, 0.5, 0.75, 1, and 2 μg/l in 900 μl H_2_O with 0.1% formic acid and 100 μl of methanol were used. The chromatographic separation was done using a Zorbax Eclipse Plus Rapid Resolution HT-C18 (100 mm × 3 mm, 1.8 μm) column on a 1,260 Infinity II HPLC (Agilent Technologies, CA, United states). Mobile phase A was H_2_O with 0.1% formic acid, while mobile phase B consisted of methanol with 0.1% formic acid. The gradient was: 90% A from 0 to 2 min, linear decrease to 40% A from 2 to 3 min, linear decrease to 0% A from 3 to 8 min, 0% A from 8 to 9 min, increase to the original condition of 90% A from 9 to 9.1 min, 90% A from 9.1 to16 min. The injection volume was 50 μl, HPLC was conducted under a constant flow rate of 0.4 ml/min and column temperature kept constant at 30°C.

The HPLC system was coupled to a QTRAP 6500+ MS/MS with linear ion trap (AB Sciex, United Kingdom). Analysis was performed in multiple reaction monitoring mode (MRM) with positive ion mode and electrospray ionization (ESI). Ion source parameters were curtain gas CUR = 30 psi, ion source gas 1 GS1 = 60 psi, ion source gas 2 GS2 = 60 psi, ion spray voltage IS = 5,000 V, collision gas CAD = medium and temperature TEM = 450°C. Dwell time was 40 ms. MRM transitions and optimized MS parameters can be found in Supplementary Table S2. All samples and standards were measured in three technical replicates.

### DNA extraction and 16S rRNA gene amplicon sequencing

For DNA extraction, one filter per sample was halved and then cut into smaller slices. Each half of the filter was placed into a separate 2 ml tube containing approx. 0.15 g of 1 mm diameter zirconium beads (Carl Roth, Germany) and 200 μl BE buffer (Macherey Nagel, Düren, Germany). After agitating for 20 s at 4 m/s (FastPrep 24, MP Biomedicals, Germany), tubes were centrifuged in a bench-top centrifuge (neoLab, Germany) and the liquid phase from both tubes was pooled. Additional 100 μl BE buffer were added to the tubes containing filters, vortexed and centrifuged. The liquid phases were combined to get maximum DNA yield. DNA extraction was further conducted following the manufacturers protocol (NucleoSpin Microbial DNA Kit, Macherey Nagel, Düren, Germany). DNA extracts were used for quantitative PCR (qPCR), as well as for sequencing of 16S rRNA gene amplicons and *intI1* gene cassettes.

To determine the diversity of the microbial community alongside the river flow, a 16S rRNA gene amplicon library was prepared for each Site from five sampling days each (Supplementary Table S1). Primers sequences for PCR-amplification of the variable region V3 of the 16S rRNA gene are given in Supplementary Table S3. For the first PCR, 1 μl of template (pure to 1:100 dilutions in ddH_2_O) was used in 12.5 μl of repliQa HiFi ToughMix (Quantabio, MA, United states), 0.75 μl of each primer 27F and 1492R (both at 10 μM) and 10 μl ddH_2_O. The PCR program was [98°C for 10 s, 55°C for 5 s, 68°C for 8 s] × 30, 8°C hold. PCR was carried out on a peqSTAR 2 × Universal gradient (VWR, PA, United states). For the nested PCR, 1.5 μl PCR product was used in 25 μl NEBNext Ultra II Q5 Master Mix (New England Biolabs, Germany), 2.5 μl of each primer 341F-TS and 518R-TS (both at 10 μM) and 20 μl ddH_2_O. The PCR program was 98°C for 30 s, [98°C for 20 s, 51°C for 30 s, 72°C for 30 s] × 35, 72°C for 30 s, 8°C hold. All PCR products were cleaned using 0.9x AmpureXP Beads (Beckmann Coulter, CA, United states) according to the manufacturers protocol and re-dissolved in 25 μl of 5 mM TE buffer. The samples were sequenced on an Illumina NextSeq 550 machine using the NextSeq 500/550 High Output Kit v2.5. For data analysis with the software package QIIME 2 (Bolyen et al., 2019; RRID: SCR_021258), all fastq files were imported as paired-end sequences, primers were trimmed and reads merged using DADA2 (Callahan et al., 2016). Classification was done in QIIME 2 using the pre-trained classifier “Silva 138 99% OTU full-length sequences” (Bokulich et al., 2018; Robeson et al., 2021). Afterwards, data was exported and manually curated in R using phyloseq (McMurdie and Holmes, 2013; RRID: SCR_013080). Before further analysis, one outlying dataset was removed for Sites 1–4 (Supplementary Figure S3). Rarefaction curves before and after rarefying the reads can be found in Supplementary Figure S4. Alpha diversity was assessed by computing the Observed Richness and Shannon diversity indices. For beta diversity, a non-metric multidimensional scaling (NMDS) plot was built from Bray-Curtis dissimilarities using the R package vegan (Oksanen et al. (2022), RRID: SCR_011950). Statistical analyses were performed using Kruskal-Wallis and Dunn’s tests in R. In addition, the beta diversity differences from all Sites were measured against the pristine Site 1. To do so, we first calculated the centroid from the NMDS beta diversity plot for all Sites and then calculated the distance between the centroid of each Site and the pristine Site 1. Furthermore, we applied the microbiome recovery model described by Shaw et al. (2019). In short, we used equation 1 to model the beta-diversity distance to Site 1.


(1) $$x\left(d\right)=\frac{De^{\Phi_{1}}e^{\Phi_{2}}}{e^{\Phi_{2}}-e^{\Phi_{1}}}\times \left(e^{-e^{\Phi_{1}}d}-e^{-e^{\Phi_{2}}d}\right)+\mathrm{Asym}*\left(1-e^{-e^{\Phi_{1}}d}\right)$$

Here, *d* refers to the geographical distance from each Site to Site 1 in km, *x*(*d*) is the calculated distance from the centroid in the NMDS plot to the pristine centroid, e is the Euler’s number, *D* is the magnitude of displacement, *Φ* refers to variables derived by the damping on the system and the strength of the restoring force, and *Asym* is the value where the equilibrium asymptotically tends to stabilize. A Bayesian framework was applied to fit Equation 1, using the R package rstan (Stan Development Team. 2022. Stan Modeling Language Users Guide and Reference Manual, v.2.21.7.)^2^ as described previously (Shaw et al., 2019). Aside from *d* and *x*(*d*), all variables were adjusted by the model. We used a uniformly distributed prior for *D*, uniform priors for *Φ_1_*, *Φ_2_*, and uniform distribution for *Asym*, as shown in equations 2.


(2.1) D~uniform(0,10)

(2.2) $$\Phi_{1}\sim \mathrm{uniform}\left(-0.99,0.99\right)$$

(2.3) $$\Phi_{2}\sim \mathrm{uniform}\left(-1,1\right)$$

(2.4) Asym~uniform(0,1)

We further investigated the phylotypes with > 1% frequency on a family level to identify phylotypes specific for each individual Site.

### Quantitative PCR

The absolute abundances of *sul1*, *sul2*, *intI1* and the bacterial 16S rRNA gene were quantified by SYBR Green-based qPCR with established primers (Supplementary Table S3) and three technical replicates per sample for all sampling dates and Sites essentially as described before (Adelowo et al., 2020). As calibration standards we used a 16S rRNA gene fragment from *Pseudomonas putida* KT2440, inserted into plasmid pGEM (Invitrogen) and cloned in *Escherichia coli* JM109, and purified PCR products obtained from *Citobacter* sp. strain EC35 for *sul1* and *sul2*, and from *Thauera aromatica* K172 for *intI1* (both strains from UFZ culture collection). The PCR mixture contained 6.25 μl KAPA SYBR^®^ FAST (Sigma-Aldrich, MO, United States), 4.75 μl ddH_2_O, 0.25 μl each of forward and reverse primer (both at 10 μM) and 1 μl of template. To compensate for PCR biases, 1:10 dilutions of each sample in ddH_2_O were prepared in transparent 96 Fast PCR-Plates (Sarstedt, Germany). Measurements were done on a StepOnePlus Real Time PCR System with software version 2.1 (AB Applied Biosystems, MA, United States). Absolute abundances were normalized to copy numbers/100 ml. The relative abundances of ARGs and the *intI1* gene were calculated as absolute abundance divided by absolute abundance of the 16S rRNA gene.

### Sequencing of class 1 integron gene cassettes

The long-read nanopore sequencing method (Oxford Nanopore Technology—ONT; Oxford, United Kingdom) was chosen to analyze the whole length of gene cassette inserts in the class 1 integron. The region between 5′CS and 3′CS of class 1 integron in the extracted DNA was inspected *via* PCR followed by long-read sequencing with ONT. For each Site, extracted DNA from nine different sampling dates was chosen (Supplementary Table S1). A volume of 2 μl of DNA template was used in 22.5 μl RedTaq 1.1 × (VWR, PA, United States), 0.5 μl each of primers 5′CS and 3′CS (both at 10 μM, Supplementary Table S3). The PCR program was: 95°C for 2 min, [95°C for 30 s, 55°C for 60 s, 72°C for 60 s] × 35, 72°C for 5 min. For Sites 1 and 2, no PCR product was visible in agarose gel electrophoresis. Therefore, these samples were not processed further. For Sites 3 to 6, band patterns observed in agarose gel were identical between all sampling dates, hence replicates were pooled before the clean-up step. PCR products were cleaned with 1.8× AmpureXP Beads, re-dissolved in 25 μl 1 mM Tris buffer and prepared for ONT sequencing using the Rapid Barcoding kit (ONT, United Kingdom) with AMPure XT beads (Beckman Coulter, CA, United States) following the manufacturer’s protocol. Flongle flow cells (ONT, United Kingdom) were loaded with 15 fmol of the total sequencing library after priming with the Flongle sequencing expansion kit (ONT, United Kingdom) following the manufacturer’s protocol. ONT sequencing was carried out on a MinION MK1b with MinKNOW software (19.06.8) for 24 h with standard settings applied. Base-calling and de-multiplexing for converting ONT fast5 to fastq files were conducted using Guppy (v3.6.0; ONT, United Kingdom). Data analysis was handled in Geneious Prime (2022.0.1, Geneious, New Zealand). Barcodes were removed by trimming 110 bp on the 5′end of each read. The Comprehensive Antibiotic Resistance Database CARD (Alcock et al., 2020) was used as a reference database from which available ARG nucleotide sequences were imported. Furthermore, we imported the sequences of 5′CS and 3′CS from the INTEGRALL database (Moura et al., 2009). All trimmed reads were then annotated from the in-house reference database choosing standard settings (“Index Length” 10 and “Best Match Criteria” 75%) and a sequence similarity of 70%, to account for the error rate of ~ 10–15% of nanopore sequencing. Non-annotated reads were checked manually using blastn with standard settings. Reads with query coverage < 40% were defined as “low quality or potential artefact” and removed (7.7% of total reads). For a graphical overview of the whole workflow, refer to Supplementary Figure S5.

## Results

### Physicochemical parameters

This study was carried out in the winter season 2020/21. The average water temperature at sampling Sites varied from 3.3°C at Sites 1 and 2 located upstream from the WWTP discharge point to 10.7°C at Site 3, the WWTP effluent, and 5°C at downstream Sites 4 to 6. The pH value at Sites 1 and 2 was on average 8.3 and 8.0, respectively. The WWTP effluent (pH 7.0) only slightly altered the pH of the river at downstream Sites 4, 5 and 6 to an average of 7.7. Nitrate concentration upstream the WWTP effluent ranged between 0.9 to 3.9 mg/l (Site 1) while downstream varied between 2.3 and 3.8 mg/l (Site 5). The nitrate concentration in the WTTP effluent was on average 3.9 mg/l, and did not affect significantly the nitrate concentration in the river (Supplementary Figure S6). The nitrate concentration in the Holtemme river were similar to those reported previously for the winter season (Brase et al., 2018), indicating that there was no substantial pollution of river water by agricultural runoff. Ammonium concentrations were low in the WWTP effluent throughout the sampling period (0.016–0.044 mg/l).

**Figure 2:**
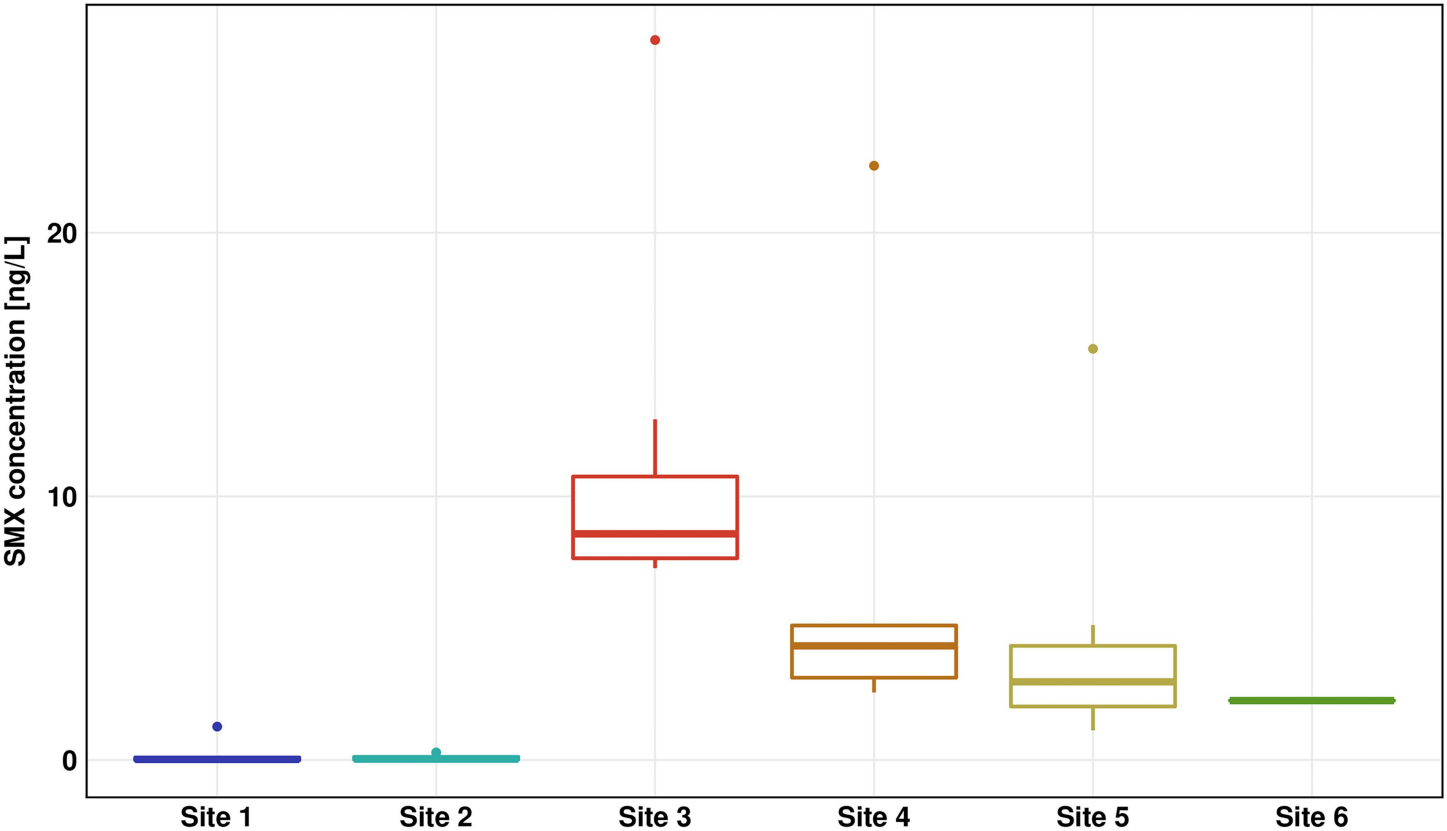
Sulfamethoxazole (SMX) concentration for all six sites measured by high performance liquid chromatography–mass spectrometry (HPLC-MS)/mass spectrometry (MS). The results of seven replicates are shown using box-whiskers-plots with the median represented by a horizontal line. Colors are representing the different sites (Site 1 in blue, Site 2 in turquoise, Site 3 in red, Site 4 in orange, Site 5 in yellow, and Site 6 in green).

The concentration of SMX was highest in the WWTP effluent (Site 3) with a median concentration of 8.6 ng/l and a peak value of 27.3 ng/l (Figure 2), which was in the low range of previous SMX quantifications in effluent of other WWTPs (Carvalho and Santos, 2016; Paumelle et al., 2020). Downstream Sites 4–6 showed a steady decrease of SMX concentrations reaching below 3 ng/l at Site 6. The SMX concentration at Site 4 was about 35% of the SMX concentration at Site 3. Thus we estimate that the wastewater contributed approximately one third to the river water in the immediate vicinity (200 m downstream), which fits well with calculations done by Krauss et al. (2019), who estimated a fraction of wastewater of 27% in the Holtemme river. SDZ and SMZ concentrations were likewise highest in the WWTP effluent, however at considerably lower concentrations than SMX with median values < 1 ng/l (Supplementary Figure S7). Previously, SMX and SMZ as well as sulfapyridine were quantified ~ 1 km downstream from our Site 4. Median SMX concentrations were about double than those in our study, while SMZ was not detectable. Sulfapyridine, which was not measured in our study, was found at a median concentration of 65.3 ng/l (Tousova et al., 2017).

### Microbial community structure

**Figure 3:**
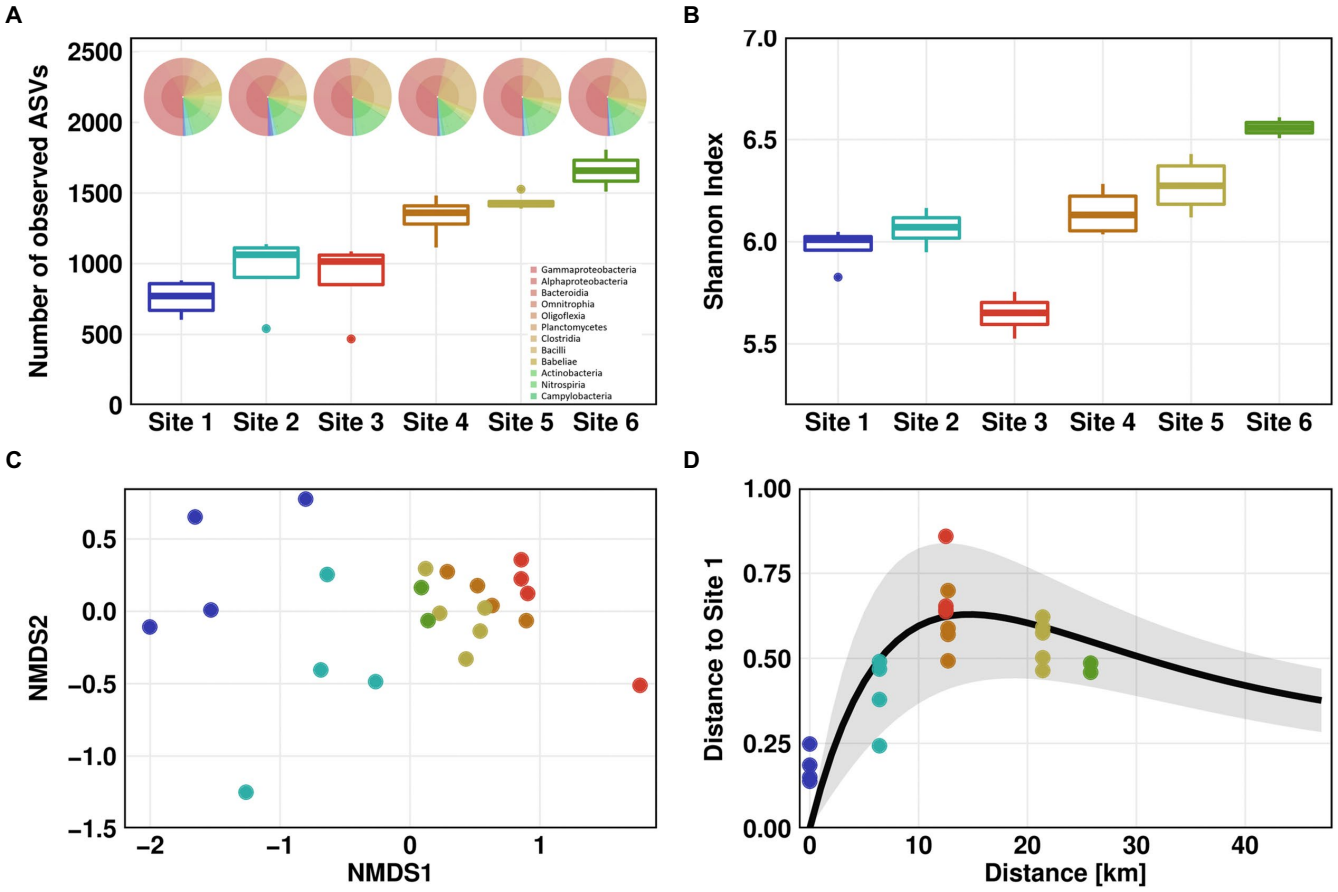
Microbial community structure. Microbial community alpha diversity based on 16S rRNA amplicon sequencing data using Observed **(A)** and Shannon measurement **(B)**. KRONA charts showing microbial community structure on genus level are displayed on top of **(A)** for February 26th, 2021. Microbial community beta diversity based on 16S rRNA amplicon sequencing data, ordination by non-metric multidimensional scaling (NMDS) of Bray–Curtis dissimilarities **(C)**. Impulse model showing the displacement from the pristine state at Site 1 **(D)**. For Sites 1–4, one outlier was removed in each dataset before analysis.

Microbial community profiling by 16S rRNA gene amplicon sequencing showed the lowest species richness according to the number of observed amplicon sequence variants (ASVs) for Site 1, with an average of 757. This value was constantly increasing along the flow path up to a number of 1,658 ASVs for Site 6 (Figure 3A). The increase in species richness was highest at Sites 3 to 4 suggesting a mixture of distinct microbial communities from WWTP and river waters. The Shannon index estimates the entropy of a system by combining species richness and evenness (Allaby, 2009). Comparable to the number of observed ASVs, the Shannon index of the river communities (excluding Site 3) was constantly increasing from Site 1 (Shannon index 6) to Site 6 (Shannon index 6.6). The average Shannon index was lowest in the wastewater effluent with a value of 5.6. (Figure 3B).

We compared the microbial diversity at all Sites using NMDS of Bray-Curtis dissimilarities. A clear separation was observed between the pristine Sites 1 and 2 on one hand, and the WWTP-impacted Sites 3 to 6 on the other hand (Figure 3C). Furthermore, downstream the discharge point of WWTP, beta diversity tended to return to the pre-discharge state. When comparing Bray-Curtis dissimilarities to those of Site 1, the differences in beta diversity were significant (Dunn’s test, *p* ≤ 0.05). Compared to Site 2, differences in beta diversity were significant for all Sites except Sites 5 and 6 (Supplementary Figure S8). These results indicate that microbial community partially recovered to its pristine state after discharge of wastewater into the river. To test this hypothesis, we visualized the microbial community’s recovery after discharge of WWTP effluent into the pristine river using a microbiome recovery model (Shaw et al., 2019). The model also showed that the microbial community was recovering after wastewater discharge yet did not fully revert to its original composition (Figure 3D).

The analysis of the taxonomic composition revealed a coherent picture of a river system with a gradient from pristine to effluent-impacted water. The family of *Caulobacteraceae*, which include many oligotrophic members, was the most abundant phylotype at pristine Site 1. Other highly abundant phylotypes at this Site were affiliated with the *Comamonadaceae* and *Sphingomonadaceae*, typical inhabitants of natural river ecosystems. Phylotypes specific for Site 1 belonged to the *Caulobacteraceae*, *Nocardiaceae* and *Rhizobiales*. The increased occurrence of phylotypes associated with the *Clostridia* taxonomic class, mainly members of *Ruminococcaceae* and *Lachnospiraceae* family, indicates a slight urban impact at Site 2, with *Flavobacteriaceae* being a phylotype specific for Site 2. Most abundant phylotypes in the effluent at Site 3 were affiliated with the genus *Trichococcus*, which is frequently found in other WWTPs (Kristensen et al., 2020). The families specific for Site 3 were *Rhodobacteraceae*, *Diplorickettsiaceae* and *Saprospiraceae. Peptostreptococcaceae* related phylotypes were detected at the downstream Sites and could not been found upstream from the discharge point. The abundance of phylotypes associated with *Comamonadaceae* and *Sphingomonadaceae* was gradually increasing downstream the WWTP discharge point (Supplementary Table S4).

### Antibiotic resistance gene abundance

**Figure 4:**
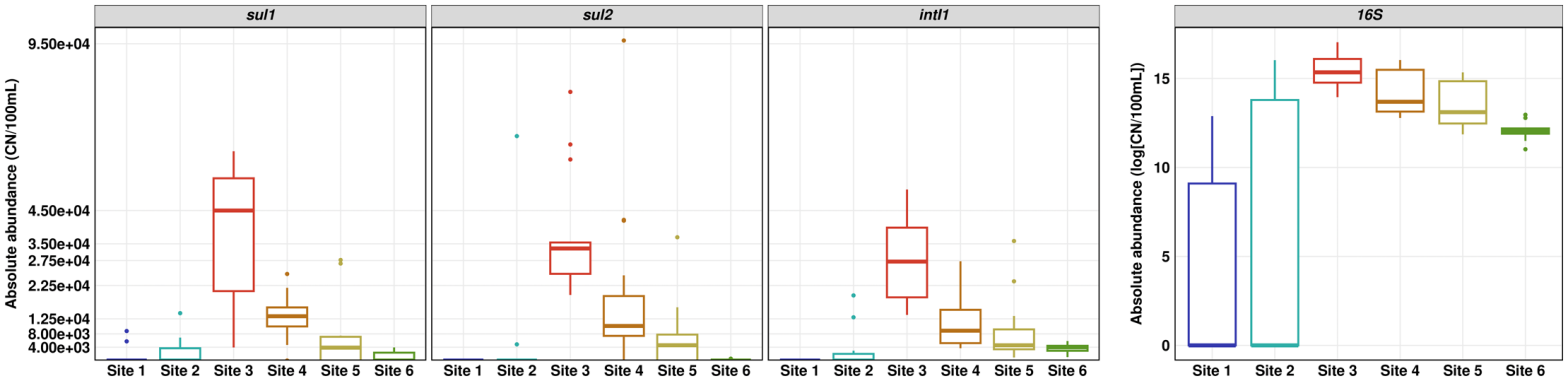
Absolute abundance of *sul1*, *sul2*, *intI1* and 16S rRNA gene determined by quantitative real-time PCR. Number of replicates (*n*) = 15.

The absolute copy numbers of the 16S rRNA gene per 100 ml did not exceed 9 × 10^6^ in river water and 2.5 × 10^6^ in the WWTP effluent. In general, the 16S rRNA gene as well as *intI1*, *sul1* and *sul2* showed the same trends with low copy numbers for pristine Site 1 and the slightly impacted Site 2, highest copy numbers in WWTP effluent and decreasing copy numbers downstream the WWTP discharge point (Figure 4). The decrease in absolute abundance of the 16S rRNA gene from Sites 3 to 4 was ~ 38%, which is in line with the decrease of the SMX concentration and supports the calculated dilution factor of WWTP effluent discharged into the river water of about one third.

**Figure 5:**
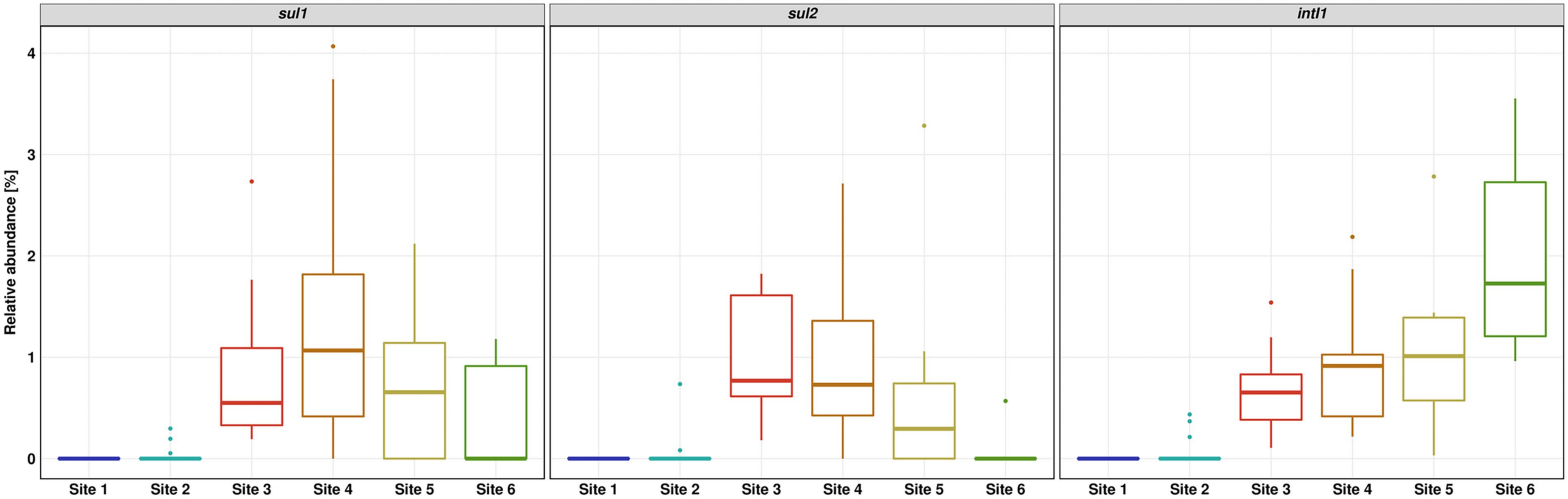
Relative abundance of *sul1*, *sul2* and *intI1* over 16S rRNA gene determined by quantitative real-time PCR. Number of replicates (*n*) = 15.

The relative abundance of *sul1* and *sul2* vs. 16S rRNA showed a similar pattern to the absolute gene abundances and measured concentrations of SMX. Surprisingly, the relative abundance of *intI1* was continuously increasing downstream the WWTP discharge point. The increase in relative abundance is statistically significant (Dunn’s test, *p* ≤ 0.05) when comparing Site 6 to Sites 3 and 4 (Figure 5).

### Gene cassette inserts

The long-read sequencing generated 340 to 684 reads per Site, with about 92% retained for further analysis after quality check. Sequence analysis revealed a high percentage of class 1 integrons without inserts in the variable region, ranging from 23% at Site 3 and up to 36% at Site 6. An even higher percentage of reads (65, 57, 64 and 63%, respectively) carried gene cassette inserts without known function. These were either not annotated previously or consisted of non-coding DNA fragments. According to GenBank queries, most of these sequences have been found previously in plasmids or integrons. Comparative analyses of sequence alignments from each individual Site revealed a decreasing trend in diversity from Sites 3 to 6.

**Figure 6:**
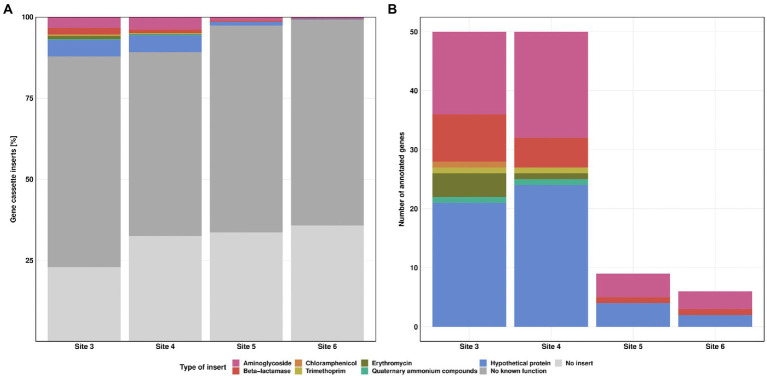
Class 1 integron gene cassette inserts. Relative frequency of insert types for each Site **(A)**. Absolute counts of annotated gene cassette inserts for each Site **(B)**. Resistance against streptomycin (pink), beta-lactamase (red), chloramphenicol (orange), trimethoprim (yellow), erythromycin (green) and quaternary ammonium compounds (turquoise). “No known function” (dark grey) refers to reads without known annotation in GenBank database. “No insert” (light grey) refers to reads where 5’CS and 3’CS were directly linked, without a variable region in between.

During the class 1 integron sequence analysis, 7.3, 6.3, 1.5 and 0.6% of the reads could be annotated as ARGs for the Sites 3–6, respectively (Figure 6A). Annotated genes showed different types of ARGs, conferring resistance against aminoglycosides, beta-lactams, chloramphenicol, trimethoprim, erythromycin and quaternary ammonium compounds. In addition, we also identified 4.9, 4.6, 1.2, and 0.3% genes for hypothetical proteins, whereby seven out of eight hypothetical proteins were previously reported in integron gene cassettes from hospital effluents (Stalder et al., 2014).

The highest diversity in inserts was observed at Site 3 with eight different types of ARGs, followed by Site 4 with seven different types. The number of annotated genes was similar for Sites 3 and 4. Sites 5 and 6 showed a clear decrease in both, diversity and number of ARGs. We detected streptomycin, beta lactamase resistance genes, and genes coding for hypothetical proteins for these Sites (Figure 6B). Multiple gene cassettes in one integron were identified only at Site 3, with one read each containing *bla*_BEL-1_|*qacL* and *aadA23*|*bla*_OXA-4_. For streptomycin resistance, we detected mainly *aadA2*, *aadA11* and *aadA6*/*aadA10*. In addition, we also found *aadA*, *aadA1*, *aadA3*, *aadA4*, *aadA6*, *aadA7*, *aadA22*, *aadA23*, *aadA24*, and *aadA25*. Most of the resistance genes against beta-lactam antibiotics were of the *bla*_OXA_ type (*bla*_OXA-2_, *bla*_OXA-4_, *bla*_OXA-10_, *bla*_OXA-33_, *bla*_OXA-36_, *bla*_OXA-129_, *bla*_OXA-392_, and *bla*_OXA-824_, each of them found at most once per Site). For the *bla*_BEL_ type we found the *bla*_BEL-1_ and *bla*_GES-11_ gene. For erythromycin resistance we detected only *ereA2*. Furthermore, we also found *cmlA5*, *dfrA1*, *dfrA7* and *qacL* genes. Of particular concern are *bla*_OXA-4_, *bla*_OXA-10_, *bla*_GES-11_, *cmlA5* and *dfrA1* as they are clinically relevant and have the greatest potential to contribute to multidrug resistance in pathogens. The *dfrA1* gene is listed as high-risk ARG by the World Health Organisation (Zhang et al., 2021). None of these high-risk genes was detected at the downstream Sites 5 and 6. Detailed information regarding the gene cassette inserts can be found in Supplementary Table S5.

## Discussion

Wastewater treatment plants (WWTP) effluents are key source of antibiotics and ARGs in riverine ecosystems (Harnisz et al., 2020; Hutinel et al., 2021; Larsson and Flach, 2022). Currently, there are conflicting results about the extent to which such contaminants spread over long distances and affect microbial communities at downstream Sites (Lee et al., 2021; Wang et al., 2021). A better understanding of the fate of ARGs in rivers will be helpful to regulatory bodies in deciding where and how best to act to curtail the spread of antibiotic resistance through environmental compartments (Vikesland et al., 2017).

Overall, our results revealed a coherent picture regarding the pollution pattern in the investigated stretch of the Holtemme river, showing the highest SMX concentration and abundance of sulfonamide resistance genes *sul1* and *sul2* in the WWTP effluent and at the nearby downstream Site 4, with a steep decrease at further downstream Sites located at 8 and 13 km distance from the discharge point. The observed pollution pattern is in line with previous studies conducted at the Holtemme, that focused on monitoring anthropogenic micropollutants in the river (Krauss et al., 2019; Beckers et al., 2020; Švara et al., 2021). We found no evidence that SMX at ambient low concentrations impacted the abundance of ARGs *sul1* and *sul2* in the river water. Most likely they shared the same source, in this case the WWTP (Larsson and Flach, 2022). Lee et al. (2021) have drawn similar conclusions, arguing that the majority of wastewater-origin resistance determinants are not persistent in rivers after discharge from WWTP due to dilution as well as additional removal mechanisms. Concerning the fate of SMX, we hypothesize that mainly physicochemical processes such as dilution, sedimentation and sorption were responsible for the concentration decrease with increasing distance to the WWTP discharge point (Luo et al., 2010).

The changes in microbial community structure reflected the pollution pattern in the Holtemme. The microbiome recovery model provided quantitative support for this ecological assumption. A pairwise diversity comparison helped identify the individual difference in the microbiome state. However, it could not provide general information on the microbiome dynamics. Our impulse model, trained based on the work from Shaw et al. (2019), gave additional insight into the long and short-term impact of wastewater treatment on the evolution of the aquatic microbiome diversity. The result of our microbiome recovery model showed the recovery of the riverine microbial community through a transition state. Both, microbial community analysis as well as our model consolidate the hypothesis that microorganisms from the wastewater may not actively thrive in the riverine environment over a more extended period, suggesting a high probability of the natural river community to recover from the displacement by effluent discharge. At the same time, it is important to notice that the community seems to be stabilizing to a different state than before the disturbance. The die-off of wastewater derived microorganisms in the river water might also explain the decrease in ARG abundance. Further investigations are needed to understand potential causal relations between the apparent stabilization of the microbial community and ARG prevalence in riverine hosts (Spencer et al., 2016). We would like to note, that our last sampling Site 6 was just upstream from the city of Halberstadt, with its own WWTP discharging. Consequently, it is likely that the microbial community structure in the Holtemme river downstream from the first WWTP will never return to its original composition. On the contrary, further WWTP effluent discharge will continuously alter the microbial community structure.

The decrease in ARG abundance downstream the WWTP, which we followed closely for *sul1* and *sul2*, was also observed for the class 1 integron. Furthermore, the diversity of gene cassette inserts was decreasing with increasing distance to the discharge point. It seems unlikely that host species carrying larger class 1 integrons were outcompeted due to the higher metabolic burden (San Millan and Craig MacLean, 2022). This suggests that some of the inserts were excised by the *intI1* gene product, potentially due to higher integrase activity triggered by cellular stress (Guerin et al., 2009; Strugeon et al., 2016; Knecht et al., 2022). Due to the missing selective pressure from high antibiotic concentrations, the loss of gene cassette inserts did not result in an evolutionary disadvantage, leading to an overall decrease in class 1 integron inserts. To our knowledge, little is known about the underlying mechanisms which are driving the integrase to either insert or excise DNA fragments from the gene cassette.

We detected some genes with high clinical relevance near the discharge point of the WWTP, like *bla*_OXA-4_, *bla*_OXA-10_, *bla*_GES-11_, *cmlA5* and *dfrA1* (Zhang et al., 2021), but none of them was found at the downstream Sites. Aminoglycoside and beta-lactam resistance genes were the most prevalent gene cassette inserts in the class 1 integron, consistent with a recent study by Yang et al. (2021), as well as a survey of the distribution of integrons in sequenced genomes (Zhang et al., 2018). Meanwhile trimethoprim resistance, which is also frequently detected in comparable studies (Wu et al., 2012; Adelowo et al., 2018; Chaturvedi et al., 2021a), was rare at our study sites. These findings highlight the diversity and complexity of class 1 integron arrays depending on environmental conditions as well as facilities located upstream from the WWTP (e.g., hospital, lifestock). Despite the high sequencing error rate, nanopore sequencing of the clinical class 1 integron can improve our understanding of environmental dynamics at comparably low cost and with sufficient sensitivity and sequencing accuracy (Kasuga et al., 2022). We anticipate that this approach will facilitate future studies on mobilized ARGs and their dissemination through the class 1 integron.

Considering the pollution pattern of the Holtemme, the application of *intI1* as biomarker for anthropogenic pollution, and the frequent association of *intI1* and *sul1* as part of the class 1 integron, the continuous increase of *intI1* relative abundance downstream the WWTP was surprising (Gillings et al., 2015; Zhang et al., 2018). Recently, Lee et al. (2021) investigated two rivers in Switzerland up- and downstream from their respective first WWTP and observed an occasional increase in the abundance of *intI1* that did not match the pollution level of the river, but they also measured a parallel increase in the abundance of *sul1*. They hypothesized that either additional sources or an in-system growth could be plausible causes. Similarly, a study on treatment wetland found an increase of *intI1* and *sul1* that occurred independent of the presence of sulfonamides (Knecht et al., 2022). In our study, the continuous increase of *intI1* relative abundance may result either from an intracellular multiplication of the gene or from the proliferation of the host microorganisms within the riverine microbial community. Interestingly, Koczura et al. (2016) likewise observed a higher relative abundance of *intI1* in river water during the cold season, which did not correlate with the abundance of *sul1*.

As our study sites are near agricultural fields, it could be hypothesized that field run-offs from agricultural lands may have introduced a variety of new phylotypes and therefore *intI1* genes to the river system. The same accounts for small tributaries of the Holtemme. These hypotheses are however not supported by the diversity analyses (Figure 3), which did not reveal a sudden emergence of new phylotypes. The potential role of other stressors, like heavy metals or a secondary source of antibiotics, as drivers for the increase in *intI1* relative abundance also appears unlikely since long-read sequencing indicated that the integrase was rather excising inserts out of the gene cassette. A remaining plausible explanation, which prompts for future investigations, is an increase in the relative abundance of *intI1* by intracellular gene multiplication.

## Conclusion

In conclusion, our monitoring study shows a partial recovery of the microbial community after wastewater discharge. SMX concentration as well as *sul1* and *sul2* abundance decreased steadily downstream from the WWTP, and the number and diversity of ARG cassettes in the class 1 integron declined. Apparently, low ng/l concentrations of sulfonamides did not stimulate maintenance or even proliferation of the corresponding ARGs in the river water. Nevertheless, copies of class 1 integron harboring ARGs against aminoglycosides and beta-lactams were still present at the most downstream Site 6, just before the river flows into the next city. Furthermore, the *intI1* relative abundance increased 3-fold over a course of 13 km downstream from the WWTP. Even though the underlying mechanism remains unclear, the observed increase was statistically significant. It seems that factors other than antimicrobials affect the environmental dynamics of *intI1* and potentially the class 1 integron. If this is the case, then limiting the release of ARG-harboring bacteria into the aqueous environment may be more prudent than attenuating ng/L concentrations of sulfonamides when aiming for restricting the environmental spread of antimicrobial resistance.

## Data availability statement

The datasets presented in this study can be found in online repositories. The names of the repository/repositories and accession number(s) can be found at: https://www.ncbi.nlm.nih.gov/, PRJNA858676. https://figshare.com/; https://doi.org/10.6084/m9.figshare.20310003.v1.

## Author contributions

NM, JM, and HHR conceived the study and the experimental design. SH and GW performed the sampling. SH executed the sample preparation, solid phase extraction (SPE), HPLC-MS, qPCR, and preparation for sequencing. FM supported with chemical analysis. SH, JK, and UR performed the microbial community analysis. SH, JM, and NM wrote the manuscript with contribution from all authors and all authors approved the submitted version.

## Funding

JK was supported by São Paulo Research Foundation (FAPESP; grant 2019/03396-9 and 2022/03534-5).

## Conflict of interest

HHR is employed by Isodetect Umweltmonitoring GmbH (Leipzig, Germany).

The remaining authors declare that the research was conducted in the absence of any commercial or financial relationships that could be construed as a potential conflict of interest.

## Publisher’s note

All claims expressed in this article are solely those of the authors and do not necessarily represent those of their affiliated organizations, or those of the publisher, the editors and the reviewers. Any product that may be evaluated in this article, or claim that may be made by its manufacturer, is not guaranteed or endorsed by the publisher.

## Footnotes

^1^ https://www.wahb.eu/

^2^ https://mc-stan.org
