# Corrigendum: The fate of sulfonamide resistance genes and anthropogenic pollution marker *intI1* after discharge of wastewater into a pristine river stream

**DOI:** 10.3389/fmicb.2023.1208555

**Published:** 2023-05-30

**Authors:** Sarah Haenelt, Gangan Wang, Jonas Coelho Kasmanas, Florin Musat, Hans Hermann Richnow, Ulisses Nunes da Rocha, Jochen A. Müller, Niculina Musat

**Affiliations:** ^1^Department of Isotope Biogeochemistry, Helmholtz Centre for Environmental Research, Leipzig, Germany; ^2^Department of Environmental Microbiology, Helmholtz Centre for Environmental Research, Leipzig, Germany; ^3^Department of Molecular Biology and Biotechnology, Faculty of Biology and Geology, Babeş-Bolyai University, Cluj-Napoca, Romania; ^4^Isodetect Umweltmonitoring GmbH, Leipzig, Germany; ^5^Institute for Biological Interfaces (IBG 5), Karlsruhe Institute of Technology, Eggenstein-Leopoldshafen, Germany

**Keywords:** class 1 integron, sulfamethoxazole, sulfonamide resistance, *sul1*, *sul2*, *inti1*, river ecosystem, one health

In the original article, there was an error in Figure 4 as published. The absolute abundances of *sul1, sul2, intI1* and 16S rRNA gene were calculated incorrectly.

The corrected Figure 4 and its caption “Figure 4. Absolute abundance of *sul1, sul2, intI1* and 16S rRNA gene determined by quantitative real time PCR. Number of replicates (*n*) = 15.” appears below.

**Figure 4:**
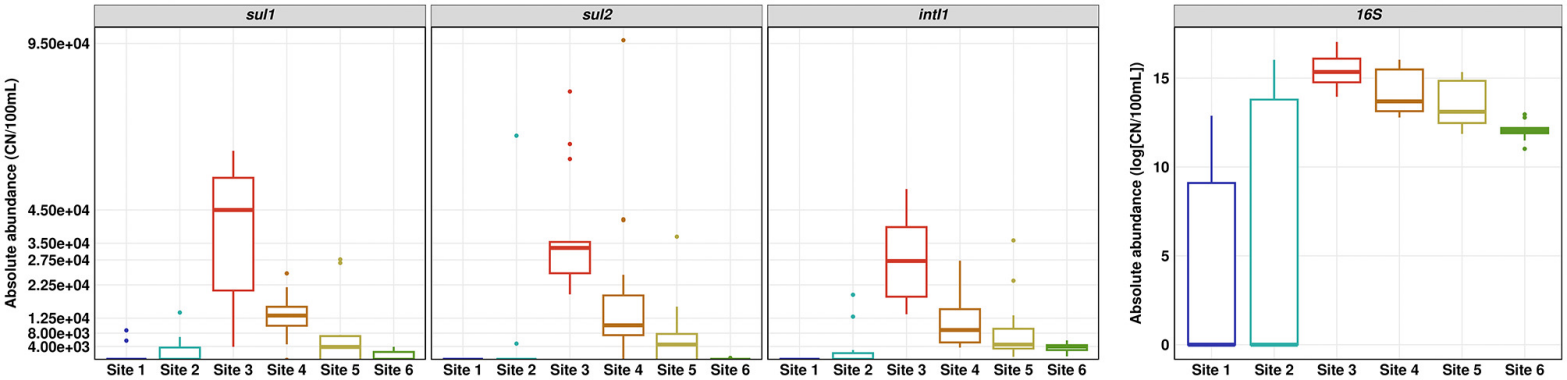
Absolute abundance of *sul1, sul2, intI1* and 16S rRNA gene determined by quantitative real-time PCR. Number of replicates (*n*) = 15.

In the original article, there was an error in the results section. The absolute abundances of *sul1, sul2, intI1* and 16S rRNA gene were calculated incorrectly.

A correction has been made to **Results**, *ARG abundance*, Paragraph 1. This sentence previously stated:

“The absolute copy numbers of the 16S rRNA gene per 100 mL did not exceed 2 × 10^5^ in river water and 5 × 10^5^ in the WWTP effluent.”

The corrected sentence appears below:

“The absolute copy numbers of the 16S rRNA gene per 100 mL did not exceed 9 × 10^6^ in river water and 2.5 × 10^6^ in the WWTP effluent.”

The author apologizes for these errors and state that this does not change the scientific conclusions of the article in any way. The original article has been updated.

