# Supplementary material for: The fate of sulfonamide resistance genes and anthropogenic pollution marker intI1 after discharge of wastewater into a pristine river stream
Source: Front Microbiol. 2023 Jan 25;14:1058350. doi: 10.3389/fmicb.2023.1058350 (PMC9907086; doi:10.3389/fmicb.2023.1058350)
Supplement: Supplementary file 1 [file Data_Sheet_1.pdf]

Supplementary Material

1 Supplementary Tables

Table S1. Sampling scheme showing the samples used for each type of analysis.

Analysis Date	HPLC-MS	qPCR	16S rRNA gene amplicon seq.	*intI1* gene cassette
7th Dec 20	✓	✓	✓	✓
4th Jan 21	✓	✓		✓
11th Jan 21	✓	✓	✓	✓
18th Jan 21	✓	✓		✓
25th Jan 21	✓	✓		✓
1st Feb 21	✓	✓	✓	✓
24th Feb 21		✓		
25th Feb 21		✓		
26th Feb 21		✓	✓	✓
27th Feb 21		✓		
28th Feb 21		✓		✓
1st Mar 21		✓		
2nd Mar 21		✓		
3rd Mar 21		✓		
4th Mar 21	✓	✓	✓	✓

Table S2. Multiple reaction monitoring (MRM) conditions for the analysis of SMX, SDZ and SMZ.

<table>
<tr><th>Analyte</th><th>Q1 (m/z)</th><th>Q3 (m/z)</th><th>Declustering Potential DP (V)</th><th>Entrance Potential EP (V)</th><th>Collision Energy CE (V)</th><th>Cell Exit Potential CXP (V)</th></tr>
<tr><td>SMX-d4</td><td>258.2</td><td>160.0</td><td>55</td><td>10</td><td>22</td><td>12</td></tr>
<tr><td rowspan="2">SDZ</td><td rowspan="2">251.1</td><td>156.1</td><td rowspan="2">45</td><td rowspan="2">10</td><td>22</td><td>14</td></tr>
<tr><td>92.0</td><td>37</td><td>10</td></tr>
<tr><td rowspan="2">SMZ</td><td rowspan="2">279.0</td><td>185.9</td><td rowspan="2">60</td><td rowspan="2">10</td><td>25</td><td>8</td></tr>
<tr><td>124.1</td><td>32</td><td>8</td></tr>
<tr><td rowspan="2">SMX</td><td>254.0</td><td>156.0</td><td>55</td><td>10</td><td>22</td><td>12</td></tr>
<tr><td>254.0</td><td>108.0</td><td>55</td><td>10</td><td>32</td><td>10</td></tr>
</table>

Table S3. Overview of primers used in this study.

<table>
<tr><th>Gene</th><th>Primers*</th><th>Sequence (5’-3’)</th><th>NCBI Ref.Seq.</th><th>Amplicon size (bp)</th><th>Reference</th></tr>
<tr><td rowspan="6">16S</td><td>27F</td><td>AGAGTTTGATCMTGGCTCAG</td><td rowspan="4">-</td><td rowspan="2">1465</td><td>Lane D. J., 1991</td></tr>
<tr><td>1492R</td><td>CGGTTACCTTGTTACGACTT</td><td>Turner et al., 1999</td></tr>
<tr><td>519qF</td><td>CCAGCAGCCGCGGTAATAC</td><td rowspan="2">410</td><td rowspan="2"></td></tr>
<tr><td>909qR</td><td>CCGTCAATTCCTTTRAGTTT</td></tr>
<tr><td>341F-TS</td><td>ACACTCTTTCCCTACACGACGCTCTTCCGATCT|CCTACGGGNGGCWGCAG</td><td>-</td><td rowspan="2">194</td><td rowspan="2"></td></tr>
<tr><td>518R-TS</td><td>GACTGGAGTTCAGACCTGTGCTCTTCCGATCT|WTTACCGCGGCTGCTGG</td><td>-</td></tr>
<tr><td rowspan="2">sul1</td><td>sul1qF</td><td>TGTCGAACCTTCAAAAGCTG</td><td rowspan="2">WP_000259031.1</td><td rowspan="2">113</td><td rowspan="2">Wang et al., 2014</td></tr>
<tr><td>sul1qR</td><td>TGGACCCAGATCCTTTACAG</td></tr>
<tr><td rowspan="2">sul2</td><td>sul2qF</td><td>ATCTGCCAAACTCGTCGTTA</td><td rowspan="2">WP_001043260.1</td><td rowspan="2">89</td><td rowspan="2">Wang et al., 2014</td></tr>
<tr><td>sul2qR</td><td>CAATGTGATCCATGATGTCG</td></tr>
<tr><td rowspan="4">intI1</td><td>5’CS</td><td>AAACGGATGAAGGCACGAAC</td><td rowspan="4">WP_000845048.1</td><td rowspan="2">variable</td><td rowspan="2">Frank et al., 2007</td></tr>
<tr><td>3’CS</td><td>ATTGCGATAACAAGAAAAAGCC</td></tr>
<tr><td>intI1qF</td><td>CGAACGAGTGGCGGAGGGTG</td><td rowspan="2">312</td><td rowspan="2">Gillings et al., 2015</td></tr>
<tr><td>intI1qR</td><td>TACCCGAGAGCTTGGCACCCA</td></tr>
</table>

*q in primer name indicates the use in qPCR

Table S4. Site-specific phylotypes and phylotypes introduced by wastewater discharge on a family level (bold face). Only phylotypes with a frequency over 1 % were considered for the analysis.

Site 1	Site 2	Site 3	Site 4	Site 5	Site 6
Caulobacteraceae	*Flavobacteriaceae*	*Rhodobacteraceae*			
Nocardiaceae		*Diplorickettsiaceae*			
Uncl. Rhizobiales		*Saprospiraceae*			
		Peptostreptococcaceae			

Table S5. List of gene cassette inserts found for each site.

Site 3	Site 4	Site 5	Site 6
aadA1[*1]<br>*aadA1*[*1]<br>*aadA1*[*1]<br>*aadA11*[*1]<br>*aadA11*[*1]<br>*aadA2*[*1]<br>*aadA2*[*1]<br>*aadA23*[*1]<br>*aadA24*[*1]<br>*aadA25*[*1]<br>*aadA4*[*1]<br>*aadA6/aadA10*[*1]<br>*aadA6/aadA10*[*1]<br>*aadA6/aadA10*[*1]<br>$bla_{\text{BEL-1}}$[*2]<br>$bla_{\text{BEL-1}}$[*2]<br>$bla_{\text{GES-11}}$[*4]<br>$bla_{\text{OXA-33}}$[*3]<br>$bla_{\text{OXA-36}}$[*3]<br>$bla_{\text{OXA-392}}$[*3]<br>$bla_{\text{OXA-4}}$[*3]<br>$bla_{\text{OXA-824}}$[*3]<br>*cmlA5*[*5]<br>*dfrA7*[*6]<br>*ereA2*[*7]<br>*ereA2*[*7]<br>*ereA2*[*7]<br>*ereA2*[*7]<br>*qacL*[*8]	*aadA1*[*1]<br>*aadA1*[*1]<br>*aadA1*[*1]<br>*aadA11*[*1]<br>*aadA11*[*1]<br>*aadA11*[*1]<br>*aadA2*[*1]<br>*aadA2*[*1]<br>*aadA2*[*1]<br>*aadA2*[*1]<br>*aadA2*[*1]<br>*aadA2*[*1]<br>*aadA23*[*1]<br>*aadA24*[*1]<br>*aadA6/aadA10*[*1]<br>$bla_{\text{BEL-1}}$[*2]<br>$bla_{\text{OXA-33}}$[*3]<br>$bla_{\text{OXA-36}}$[*3]<br>*ereA2*[*7]<br>*qacL*	*aadA2*[*1]<br>*aadA6/aadA10*[*1]	*aadA2*[*1]<br>*aadA2*[*1]<br>*aadA2*[*1]<br>$bla_{\text{OXA-36}}$[*3]

Gene family | Drug class

[*1] ANT(3'') aminoglycoside nucleotidyltransferase | aminoglycosides
[*2] BEL beta-lactamase | cephalosporins, penams, monobactams
[*3] OXA beta-lactamase | carbapenems, cephalosporins, penams
[*4] GES beta-lactamase | carbapenems, cephalosporins, penams
[*5] Major facilitator superfamily (MFS) antibiotic efflux pump | phenicol
[*6] Trimethoprim resistant dihydrofolate reductase dfr | diaminopyrimidines
[*7] Macrolide esterase | Macrolides
[*8] Small multidrug resistance (SMR) antibiotic efflux pump | disinfecting agents and antiseptics

2 Supplementary Figures

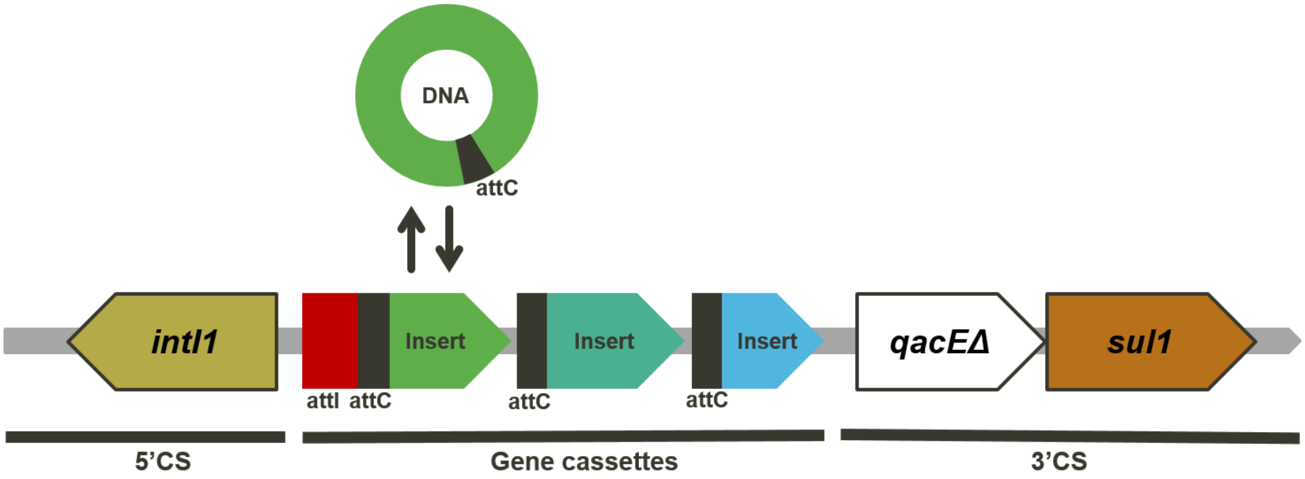

Figure S1. Structure of the Class 1 integron.

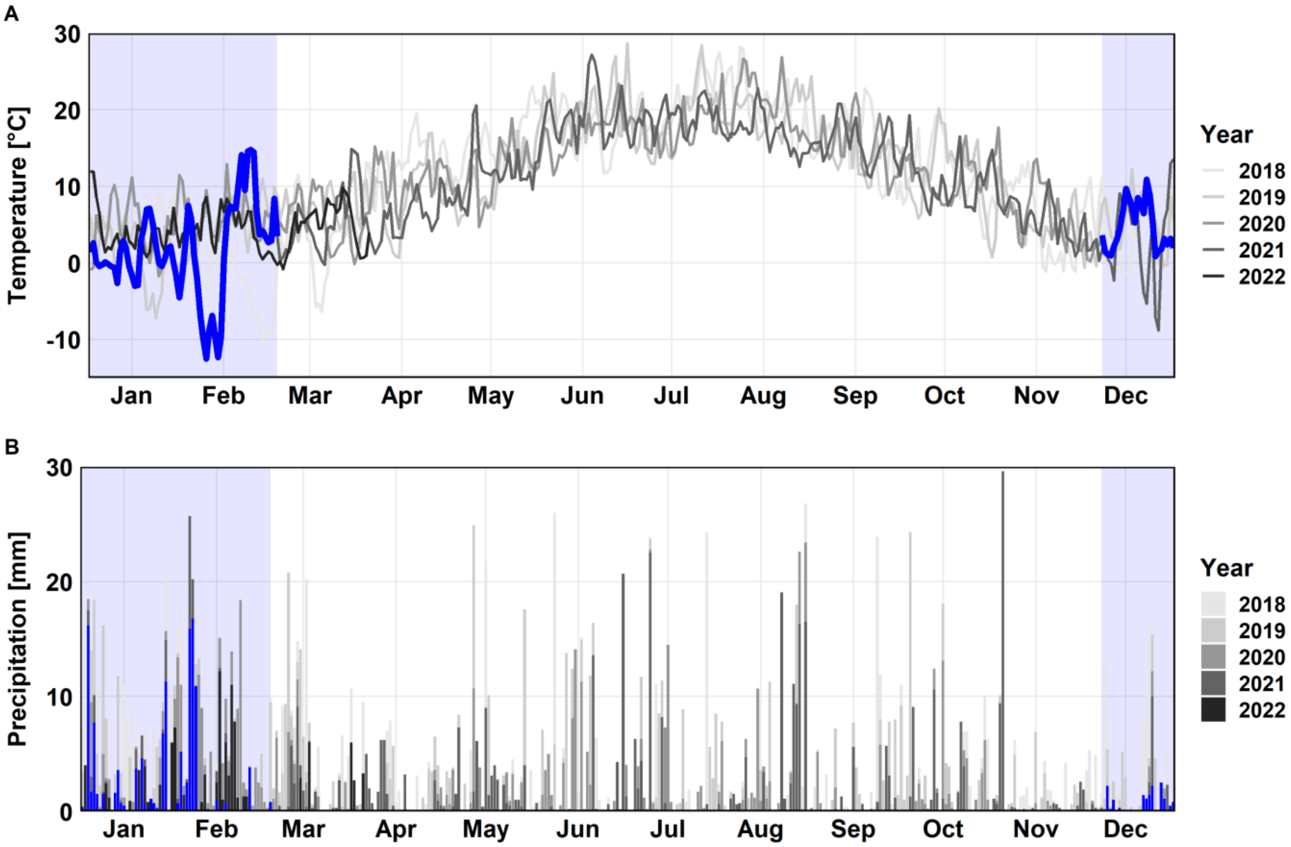

Figure S2. Temperature (A) and precipitation (B) in Wernigerode city from January 2018 until March 2022. Blue lines and bars are highlighting the data from December 2020 until March 2021. Data was received from the German weather monitoring station 5490 (https://opendata.dwd.de/).

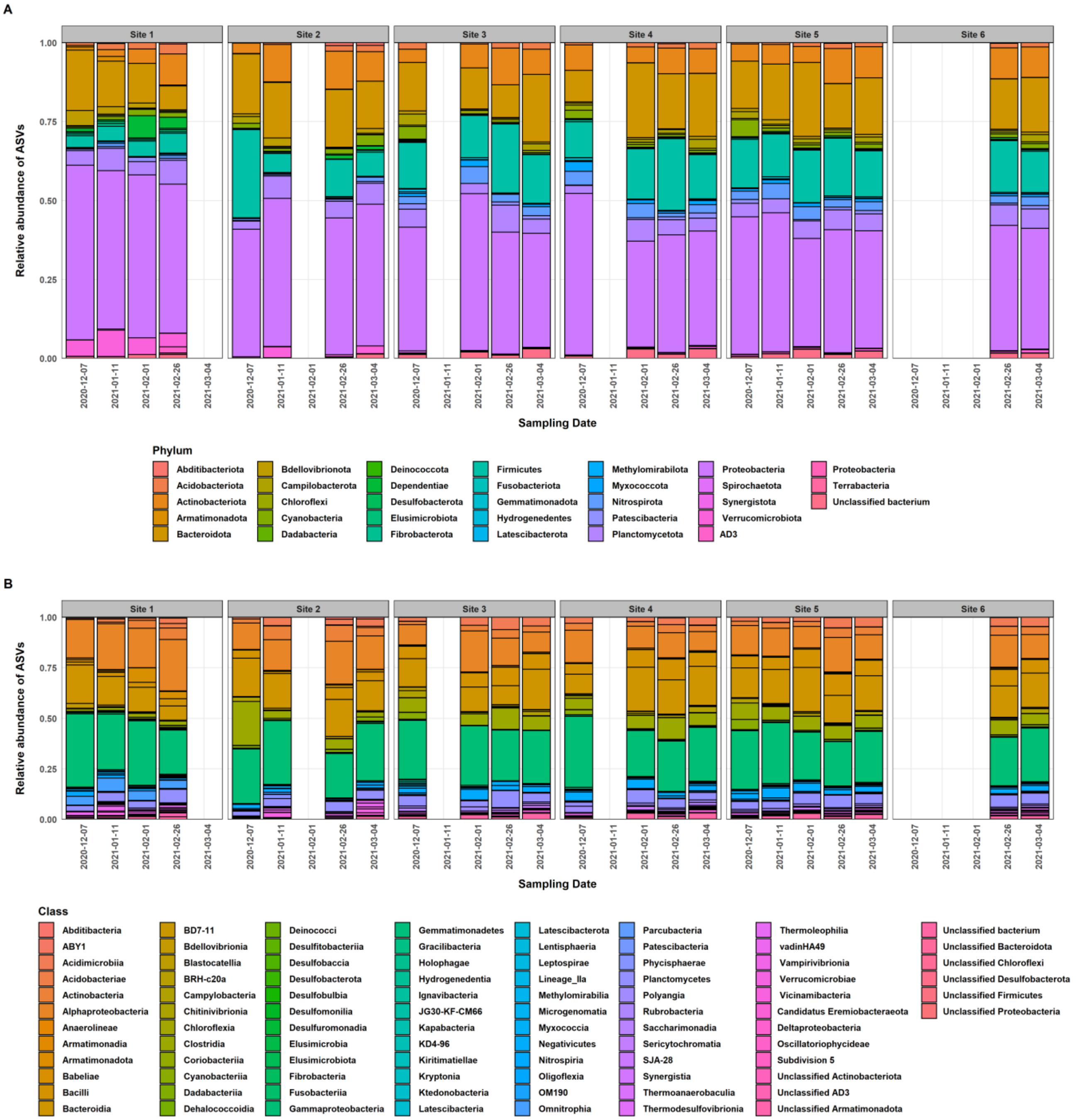

Figure S3. Microbial community structure at phylum (A) and class level (B) for each site, sorted by sampling date. For Sites 1 to 4, one outlier was removed in each data set before the analysis.

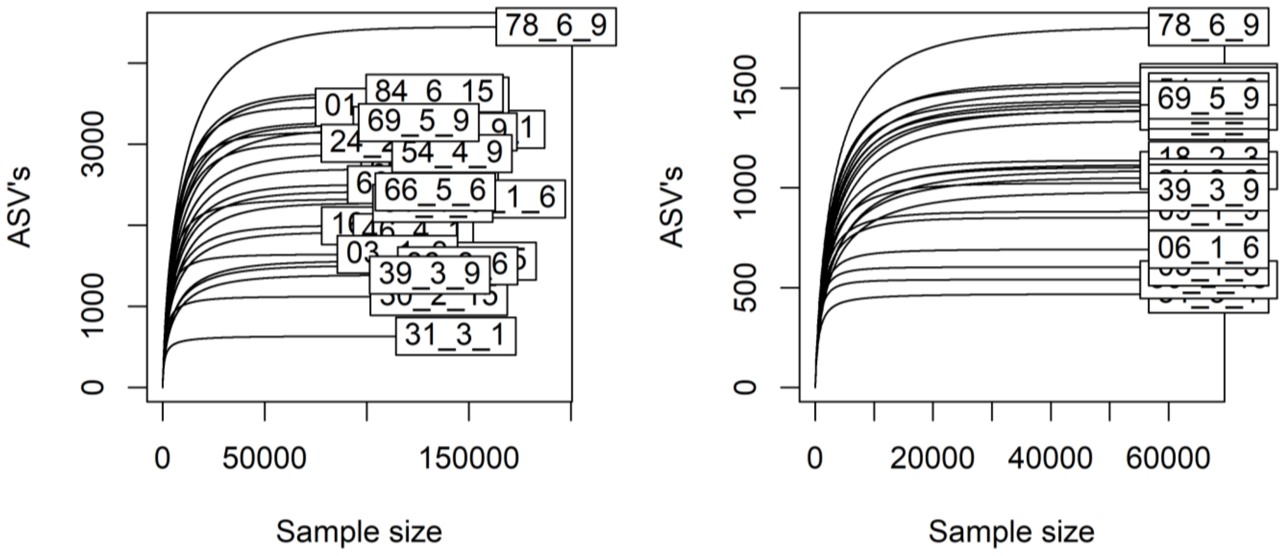

Figure S4. Rarefaction curves for 16S rRNA gene sequencing before (left) and after (right) removal of low abundance ASV's.

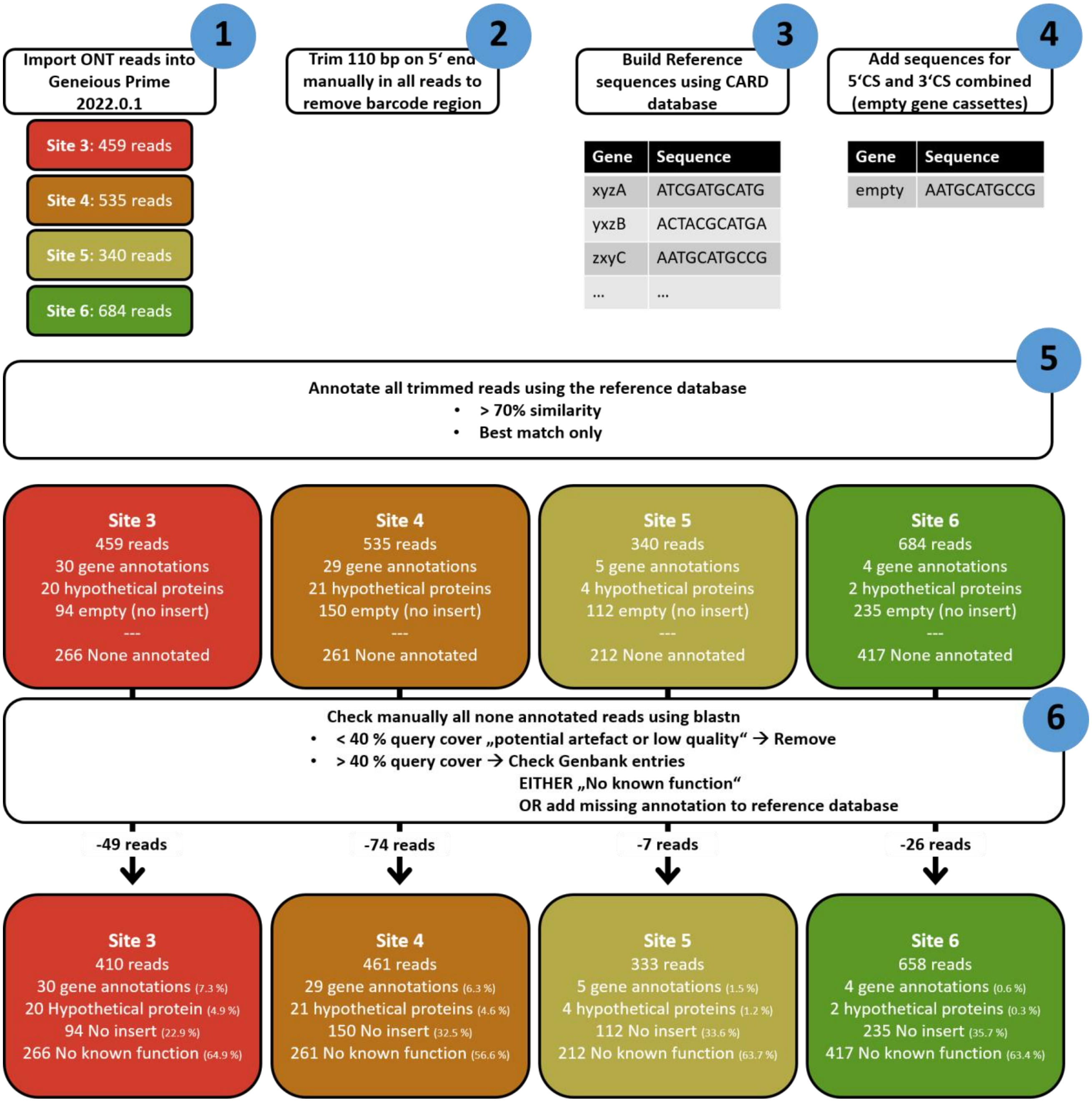

Figure S5. Workflow for the analysis of class 1 integron gene cassette inserts after ONT sequencing.

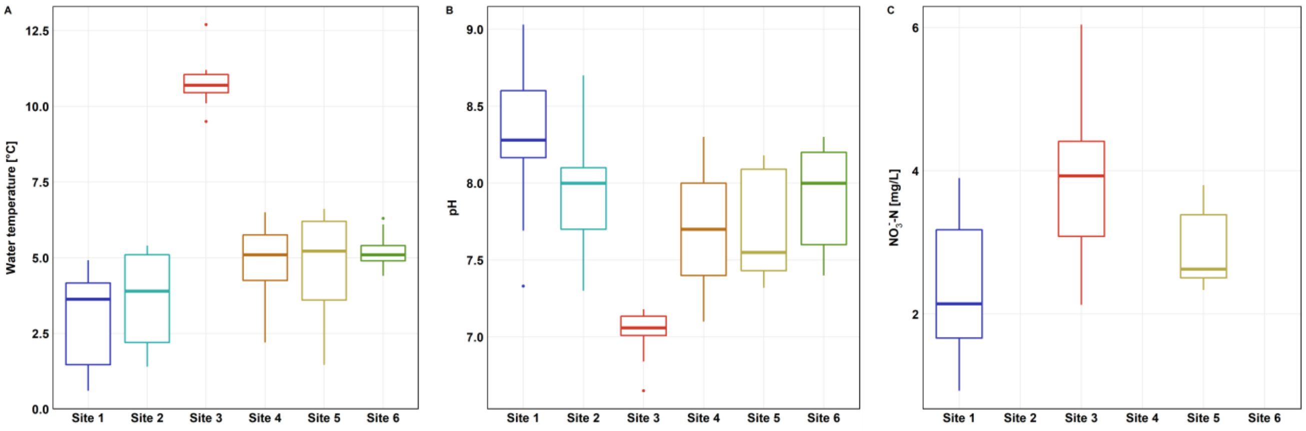

Figure S6. Physicochemical parameters measured at all Sites. Measurements were obtained from MOBICOS monitoring stations (site 1 and 5, https://www.ufz.de/index.php?en=39611), WWTP Silstedt (site 3, https://www.wahb.eu/) and manually using a SenTix41 probe (site 2, 4 and 6). Water temperature (A). pH (B) and $NO3^{-}$-N (C). Distinct colours were used to differentiate between individual Sites.

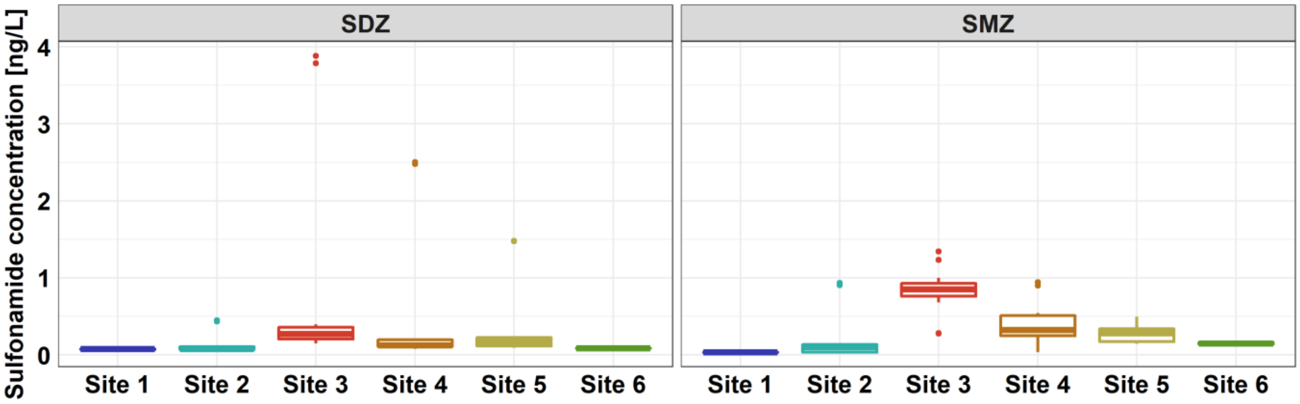

Figure S7. SDZ and SMZ concentrations for all six Sites measured by HPLC-MS/MS. The results of seven replicates are shown using box-whiskers-plots with the median represented by a horizontal line. Distinct colours were used to differentiate between individual Sites.

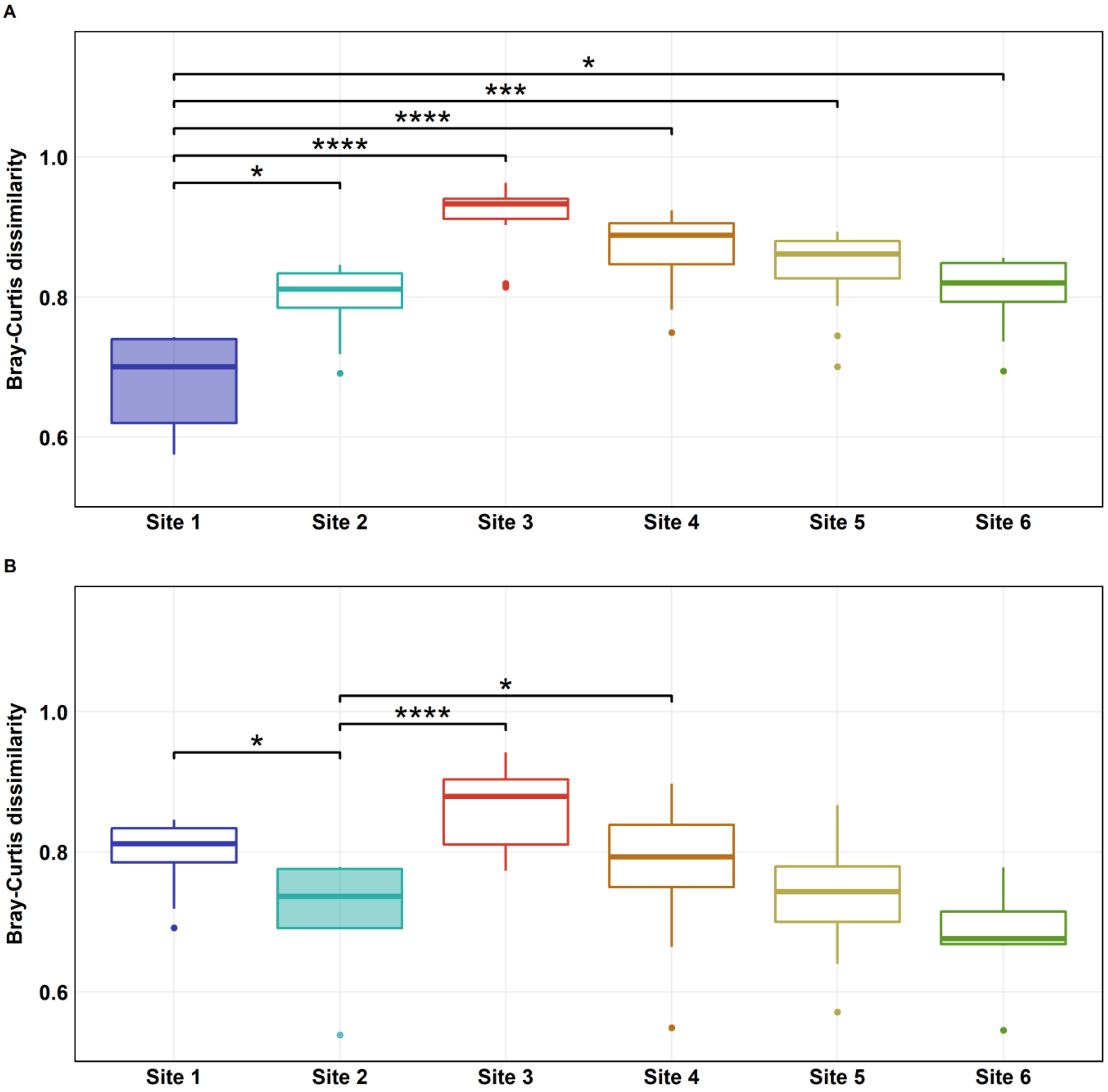

Figure S8. Bray Curtis dissimilarity in comparison to site 1 – pristine (A). Bray Curtis dissimilarity in comparison to site 2 – city (B). Significant differences between Sites are represented by asterisks (Dunn's test, * ≤ 0.05, *** ≤ 0.001, **** ≤ 0.0001). Distinct colours were used to differentiate between individual Sites.

3 References

Frank, T., Gautier, V., Talarmin, A., Bercion, R., and Arlet, G. (2007). Characterization of sulphonamide resistance genes and class 1 integron gene cassettes in Enterobacteriaceae, Central African Republic (CAR). *J Antimicrob Chemother* 59, 742–745. doi: 10.1093/jac/dkl538

Gillings, M. R., Gaze, W. H., Pruden, A., Smalla, K., Tiedje, J. M., and Zhu, Y.-G. (2015). Using the class 1 integron-integrase gene as a proxy for anthropogenic pollution. *ISME J* 9, 1269–1279. doi: 10.1038/ismej.2014.226

Lane D. J. (1991). 16S/23R rRNA sequencing. *Nucleic acid techniques in bacterial systematics*. 115–175.

Turner, S., Pryer, K. M., Miao, V. P., and Palmer, J. D. (1999). Investigating deep phylogenetic relationships among cyanobacteria and plastids by small subunit rRNA sequence analysis. *J Eukaryotic Microbiology* 46, 327–338. doi: 10.1111/j.1550-7408.1999.tb04612.x

Wang, N., Yang, X., Jiao, S., Zhang, J., Ye, B., and Gao, S. (2014). Sulfonamide-resistant bacteria and their resistance genes in soils fertilized with manures from Jiangsu Province, Southeastern China. *PLoS One* 9, e112626. doi: 10.1371/journal.pone.0112626
